# Willingness to Pay for Social Health Insurance and Its Predictors among Government Employees in Mujja Town, Ethiopia

**DOI:** 10.1155/2021/3149289

**Published:** 2021-03-04

**Authors:** Feleku Mekonnen Degie, Yeshambel Agumas Ambelie, Yared Mulu Gelaw, Getahun Fentaw Mulaw, Fentaw Wassie Feleke

**Affiliations:** ^1^North Wollo Zonal Health Department, Woldia, Amhara Regional State, Ethiopia; ^2^School of Public Health, College of Medicine and Health Sciences, Bahirdar University, Bahir Dar, Ethiopia; ^3^School of Public Health, College of Health Science, Woldia University, Woldia, Ethiopia

## Abstract

**Background:**

Social health insurance is one of the possible organizational mechanisms for raising and pooling funds to finance health services, private health insurance, community insurance, and others.

**Objective:**

The study was aimed to assess willingness to pay for social health insurance and associated factors among government employees in Mujja town, Ethiopia.

**Methods:**

An institutional-based cross-sectional study was conducted on the total sample size of 375 study respondents. A simple random sampling technique was employed. Data were entered into EPI info 7 and analyzed by Statistical Package for Social Sciences version 22.0. Multivariable logistic regression was used to identify independent predictors by controlling confounding variables. Statistical significance was declared at *p* < 0.05.

**Results:**

This study revealed that 37.6% (95% CI: 33.1%, 42.61%) respondents were willing to pay for social health insurance. In the final model, respondents who ever heard about health insurance schemes were seven times (AOR = 7.205; 95% CI: 1.385, 37.475) more likely willing to pay for social health insurance. Thos who had history of difficulty and having other source to cover medical bills were 92.6% (AOR = 0.074; 95% CI: 0.009, 0.612) and 94.6% (AOR = 0.054; 95% CI: 0.011, 0.257) less likely to pay, respectively.

**Conclusions:**

Willingness to pay for social health insurance was low. Being heard about health insurance, history of difficulty, and having other sources to cover medical bills were associated factors. Thus, it is recommended that media promotion and these factors should be considered for the successful implementation of the scheme.

## 1. Background

Social health insurance (SHI) is one of the possible organizational mechanisms for raising and pooling funds to finance health services, along with tax-financing, private health insurance, community insurance, and others [1]. Health insurance is promoted in developing countries since the 1990s to improve access to healthcare services [2]. SHI schemes are provided by governments and nongovernment organizations to its citizens, especially to low- and middle-income populations [3].

Significant proportions of people all over the world suffer and die due to a lack of access to basic healthcare services. In low- and middle-income countries alone, 150 million people suffer a health-related financial catastrophe each year, and 100 million people are pushed into poverty as a result of out-of-pocket (OOP) health expenditure [4–7].

Introducing SHI was considered as one of the most powerful risk pooling mechanisms in most developing countries to achieve universal health coverage (UHC). The UHC had been achieved in many countries in the world by establishing SHI as the country's healthcare financing mechanism [8]. It improves access to health services by removing catastrophic health expenditures by pooling funds to allow cross-subsidization between the rich and poor and between the healthy and the sick [9].

The Ethiopian government developed a health insurance strategy in 2008, and two types of health insurance have been proposed since 2010, Community Based Health Insurance (CBHI) and SHI [10, 11]. The SHI was intended to cover the employed and their family members, approximately 11% of the population (public servants, permanent employees working in private organizations, and pensioners). Enrollment in SHI is compulsory and the proposed contribution is 3% of their salary [10]. But it is impossible to force people to join SHI because it will lead to problems of low coverage, adverse selection, and fragmented risk pools [4].

The Ethiopian healthcare system is characterized by high OOP expenditure, increased healthcare needs, inability to mobilize more resources for health, and inability to fully recover costs of care incurred by beneficiaries [12]. The Ethiopian health insurance coverage of the total population is very low, representing only 7.5% [13].

Despite the government plan to fully implement SHI by 2014 [14–17], it has been repeatedly postponed, which is largely due to strong resistance from government employees. There was limited evidence on willingness to pay and associated factors for the SHI scheme in Ethiopia, the Amhara region particularly in the study area, Muja. Hence, this study was conducted to explore the willingness to pay for SHI and associated factors among government employees in Mujja town, northeast Ethiopia. The study focused on teachers, health professionals, polices, and women and child affairs employed in Mujja town.

## 2. Methods and Materials

### 2.1. Study Design, Period, and Setting

A quantitative institutional-based cross-sectional study design was carried out from February 15 to May 20/2016. Mujja town is one of the 15 North Wollo administrative woredas, which is located around 594 Km from Addis Ababa and 345 km from Bahir Dar. This is bounded by Tigray in the North, Meket, and Gubalafto woreda in the South, Kobo woreda in the East, and Lasta woreda in the West. There were around 981 employees working in a governmental institution in the town, out of which 651 and 330 were male and female population, respectively. With regard to health infrastructure; there was only one functional health center.

### 2.2. Source, Study Population, and Study Unit

The source population included individuals who were working in all government institutions in Mujja town while the study population was employees who were working in the randomly selected government institutions. The study units were employees on whom the data were actually collected.

### 2.3. Eligibility Criteria

Full-time employees working for more than six months in government institutions were included. Those employees who were working under CBHI unit were excluded.

### 2.4. Sample Size and Sampling Technique

The sample size was determined by using a single population proportion formula with the following assumptions: the proportion of willingness to pay for SHI as 74.4% [18], margin of error 0.05%, design effect 2, 95% confidence level, and 10% nonresponse rate.

(1) $$\begin{array}{c} n=\text{diff}\frac{\left(Z^{2}P\left(1-P\right)\right)}{d^{2}}=2\frac{{\left(1.96\right)}^{2}\left(0.744\right)\left(0.256\right)}{{\left(0.05\right)}^{2}}=586. \end{array}$$

After adding 10% of nonresponse rate, the final sample size will be 645.

Since the source populations were less than 10,000, adjustments were also done.

(2) $$\begin{array}{c} \text{Adjusted }n=\frac{n}{1+\left(n/N\right)}=\frac{645}{1+\left(645|/|981\right)}=390. \end{array}$$

Therefore, the final sample size after adjustment is 390.

### 2.5. Sampling Procedure

**Figure 1:**
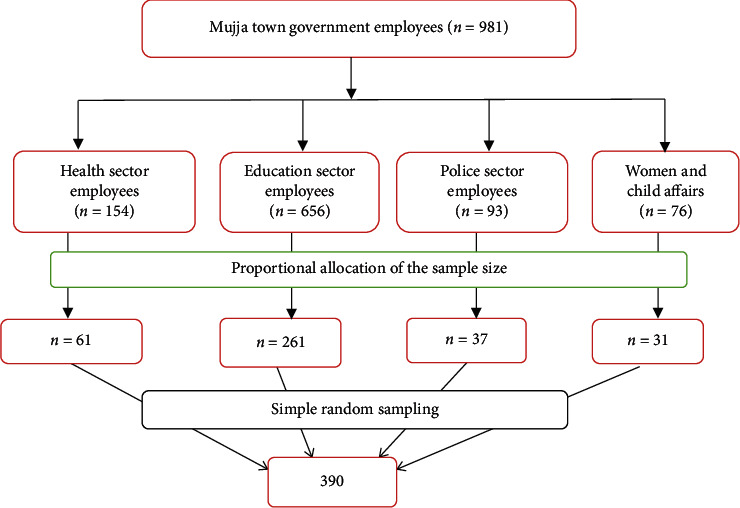
Schematic representations of the sampling procedure for willingness to pay to SHI and associated factors among government employees in Mujja town, northeast Ethiopia.

Simple random sampling technique was used. The governmental institutions included were the health sector, education sector, police, and women and child affairs. Then, the sample size was allocated proportionally for each sector, and finally, study units were selected by simple random sampling (Figure 1).

### 2.6. Data Collection Tools and Quality Assurance

Data were collected by a self-administered semistructured questionnaire on selected governmental employees. The questionnaire, developed through a critical review of relevant literature [14, 15, 17, 19–21], was composed of three parts. These were socio-demographic and economic characteristics, health status, and healthcare utilization of the individuals, exposure, and perception regarding insurance. Four degree-holder statisticians as data collectors and two BSc male public health professionals as supervisors working in the district were recruited.

The research questionnaire was prepared in the English version and translated into the local language (Amharic) and retranslated back to English to check consistency by different peoples. Before the actual data collection, the questionnaire was pretested on 10% of the calculated sample size in none selected governmental institutions of Muja town, and then necessary modification was done accordingly before the actual data collection.

Data collectors and supervisors who were fluent in the local language were recruited and two days of training was given before the actual data collection. The training was on interviewing skills, probing techniques of the SHI scheme. Continuous supervision and follow up of the data collectors were done. The collected data were handled and stored carefully and appropriately.

### 2.7. Data Management, Processing, and Analysis

Data were checked for incompleteness and inconsistency, edited, cleaned, coded, and entered into EPI info version 7 and exported to SPSS version 22.0 software for analysis. Descriptive statistics for categorical variables were presented using frequency and percentage.

Multi-collinearity among predictor variables was checked using standard error. Those variables with a standard error of two or more were considered having multi-collinearity, but no variable was found. The Omnibus test yielded a *p* value <0.0001 with Hosmer and Lemeshow goodness of fit test (*p*=0.969). Multivariable logistic regression was used to identify independent predictors of willingness to pay for SHI schemes by controlling confounding variables. Predictor variables with the *p* value <0.25 at bivariable were included into the multivariable logistic regression model. The strength of association was measured through the adjusted odds ratio at a 95% confidence interval.

### 2.8. Operational Definitions

- Willingness to pay: volunteer to pay for the proposed first bid (3% of monthly salary) of the premium. For those who did not ever hear about HI, information was given in detail about what SHI mean, its objective and payment-related and healthcare-related issues before asking them about their willingness to pay.
- Full-time employees: employees who work for more than six months in a governmental institution for their full working time.
- History of difficulty to cover medical bill(s): those who were suffering to cover the medical expense during previous illness times.
- Having other sources to cover medical bill: those whose medical expense is covered by other sources (the medical bill is not covered by their pocket money).

## 3. Results

### 3.1. Socio-Demographic Characteristics of Study Participants

A total of 375 study participants were included in this study with a response rate of 96.2%. The majority, 337 (89.9%), of the study participants were Amhara ethnic groups. Their mean (±SD) age was (30.59 ± 7.05) years and two-thirds (63.5%) of them were male. The median (±IQR) monthly income of the respondents was 2493.824 (±1203.058) ETB (Table 1).

### 3.2. Health Status and Healthcare Utilization of the Individuals

Regarding preferences of treatment-seeking behavior during illness, 265 (70.7%) of the study participants preferred governmental health facilities. Nearly two-thirds, 248 (66.1%), of respondents paid for their medical bill out of pocket and 89 (23.7%) employees' medical bill was covered by the government. Out of the total respondents, 238 (63.5%) had a history of difficulty to cover their medical bills (Table 2).

### 3.3. Exposure and Perception towards SHI

Only 54 (14.4%) of the employees have ever heard about the health insurance scheme. More than half, 215 (57.3%), of the respondents had thought as government should pay for their SHI. Near to one-fourth 92 (24.5%) of respondents believed that paying for their SHI is similar to monthly saving (Table 3).

### 3.4. Willingness to Pay for Social Health Insurance

In this study, 37.6% (95% CI: 33.1%, 42.61%) of participants were willing to pay for the suggested insurance scheme (Table 4).

### 3.5. Factors Associated with Willingness to Pay for SHI

In the final multivariable logistic regression model, respondents who ever heard about health insurance schemes were seven times (AOR = 7.205; 95% CI: 1.385, 37.475) more likely to be willing to pay as compared to those employees who never heard of social health insurance.

Respondents who had other sources to cover their medical bills were 92.6% (AOR = 0.074; 95% CI: 0.009, 0.612) less likely to be willing to pay for SHI than respondents having other sources.

Study participants who had a history of difficulty covering their medical bills were 94.6% (AOR = 0.054; 95% CI: 0.011, 0.257) less likely to pay for SHI than those who can pay for their medical bill (Table 5).

## 4. Discussion

This study showed that overall willingness to pay for SHI was 37.6% which was lower than a study done in Bahi Dar city (66.6%) [22], Mekelle city (85.3%) [23], Wolaita Sodo (74.4%) [18], and Nigeria (89.7%) [21]. This could be due to the difference in socio-demographic characteristics of study participants. But this study finding was slightly higher than other studies done in Ethiopia, Tigray region (35.5%) [24], and Addis Ababa city (28.7%, 17%) [25, 26].

This study found that these respondents who ever heard about SHI, having a history of difficulty to cover their medical bills and having other sources to cover their medical bills, were the factors statistically significantly associated with willingness to pay for SHI.

In this study, respondents who ever heard about health insurance schemes were more likely to be willing to pay as compared to those employees who never heard. This is supported with a study done in southern Ethiopia, Wolaita Sodo town [18]. This could be due to that there was no health insurance scheme (SHI or CBHI) in the town before and knowing about the benefits of health insurance is important to be willing to pay. This might be also due to having good awareness about health insurance and trust in a health insurance agency [22, 27, 28].

Those who had other sources to cover their medical bill were less likely willing to pay for SHI. This maybe due to their medical cost being already covered and they consider it as extra cost [19, 20]. This study might be also due to their higher income from other sources, which makes them able to cover their medical expense OOP money and by other sources [23, 26].

Furthermore, those who had no history of difficulty covering their medical bill were less likely willing to pay for SHI. This is consistent with a study revealed from the northern part of Ethiopia, Mekelle city [28], and southern Ethiopia, Wolaita Sodo town [18], Sierra Leone [27]. This might be due to that individuals who were unable to cover their medical bill may need risk-sharing nature of the insurance [29–31]. This might be due to the presence of abiding rule of referral system to get higher-level health services, exclusion of periodic medical checkup from SHI, poor perception of service quality, and presence of financial insecurity in government health institutions, the amount of premium contribution, and the perception that SHI will create a workload for health workers [23–25, 28].

### 4.1. Limitation of the Study

This study assessed the willingness to pay for SHI from participants who have insured and not insured sectors. Because of this, the respondents might have a higher ambition and expectation from the social health insurance before knowing the actual importance of SHI, which might have an effect on the magnitude of willingness to pay for SHI. Some variables were not measured such as wealth index and satisfaction on the quality of the healthcare services which might be predictive of willingness to pay for SHI. Another limitation of this study could be social desirability bias in which health professionals may tend to give favorable responses as they are the main stakeholders for sensitizing and implementing health-related policies. Hence, the conclusion of these findings should be interpreted considering these limitations.

## 5. Conclusion

In conclusion, this study revealed that willingness to pay for SHI was low. Being heard about health insurance, history of difficulty to cover their medical bills, and having other sources to cover their medical bill were associated factors of willingness to pay for SHI. Thus, it is recommended that media promotion for adequate awareness creation and the above factors should be considered with all employees at various levels for the successful implementation of the scheme. We recommend also other researchers to address other possible factors such as trust issues, quality services, wealth status, distance nearby health service, media coverage, and service satisfaction through various designs and methods.

## Figures and Tables

**Table 1:** Socio-demographic and economic characteristics of the government employees in Muja town, northeast Ethiopia, 2016 (*n* = 375).

Variable	Category	Frequency	Percent
Age	≤24	54	14.4
	25–29	162	43.2
	30–34	66	17.6
	35–39	41	10.9
	>40	52	13.9
	Mean ± SD	(30.59 ± 7.05)	

Sex	Male	238	63.5
	Female	137	36.5

Educational status of respondents	Below diploma	21	5.6
	Diploma	173	46.1
	First degree and above	181	48.3

Ethnicity	Amhara	337	89.9
	Others^*∗*^	38	10.1

Marital status	Married	203	54.1
	Not married	172	45.9

Occupation	Health	58	15.4
	Police	34	9.1
	Education	255	68.0
	Women and child affairs	28	7.5

Religion	Orthodox	338	90.1
	Others^*∗∗*^	37	9.9

Family size	1	101	26.9
	2-3	155	41.3
	4-5	91	24.3
	≥6	28	7.5

Monthly income (ETB)	<1400	35	9.3
	1401–2350	178	47.5
	2351–3550	114	30.4
	>3550	48	12.8
	Median ± IQR	2493.824 (±1203.058)	

^*∗*^Tigray, Oromo, and Afar.  ^*∗∗*^Muslim, Protestant, and Catholic.

**Table 2:** Health status and healthcare utilization of the government employees in Muja town, northeast Ethiopia, 2016 (*n* = 375).

Variable	Category	Frequency	Percent
Preferences of treatment seeking during illness	Governmental health facility	265	70.7
	Private health facility	78	20.8
	Traditional healers	32	8.5

Chronic disease in the family	Yes	30	8
	No	345	92

Payment management	Government	89	23.7
	Own money	248	66.1
	Borrowed	38	10.2

History of difficulty to cover medical bill(s)	No	238	63.5
	Yes	137	36.5

Confidence to afford the care they need during illness	Yes	235	62.7
	No	140	37.3

Having other source to cover medical bill	Yes	95	25.3
	No	280	74.7

**Table 3:** Exposure and perception regarding SHI of the government employees in Muja town, northeast Ethiopia, 2016 (*n* = 375).

Variable	Category	Frequency	Percent
Ever heard about SHI	Yes	54	14.4
	No	321	85.6

Who do you think should pay for insurance	Employees	19	5.1
	Employer	72	19.1
	Government	215	57.3
	Shared	69	18.5

Believe that insurance is similar to monthly savings	Yes	92	24.5
	No	283	75.5

Think insurance is a compensation if something bad happens	Yes	278	74.1
	No	97	25.9

**Table 4:** Willingness to pay for SHI among government employees in Muja town, northeast Ethiopia, 2016(*n* = 375).

Variable	Category	Frequency	Percent
WTP for SHI	No	234	62.4
	Yes	141	37.6

Reason for not willing to pay for the social health insurance scheme (*n* = 234)	I am always in good health	99	42.3
	SHI does not cover need	126	53.8
	Fear of poor implementation	9	3.9

**Table 5:** Factors associated with willingness to pay for SHI among government employees in Muja town, northeast Ethiopia, 2016 (*n* = 375).

Variable/category	WTP		COR (95% CI)	AOR (95%)
	Yes	No		
*Ever heard about SHI*				
Yes	36 (66.7)	18 (33.3)	4.114 (2.231, 7.587)	7.205 (1.385, 37.475)^*∗*^
No	105 (32.7)	216 (67.3)	1	1

*Having other source to cover medical bill*				
Yes	12 (12.6)	83 (87.4)	0.169 (0.088, 0.324)	0.074 (0.009, 0.612)^*∗*^
No	129 (46.1)	151 (53.9)	1	1

*History of difficulty to cover medical bill*				
Yes	102 (74.5)	35 (25.5)	1	1
No	39 (16.4)	199 (83.6)	0.067 (0.040, 0.113)	0.054 (0.011, 0.257)^*∗∗*^

Those variable(s) statistically associated at binary logistic regressing with *p* < 0.25 and entered to the final model were as follows: being heard about SHI, believing that insurance is similar to monthly saving, having other sources to cover a medical bill, history of difficulty to cover their medical bill, and having a chronic disease in the family.  ^*∗*^*p* value <0.001 and  ^*∗∗*^*p* value <0.005.

## Data Availability

The data used in this study are available upon reasonable request to the corresponding author.

## Abbreviations

AOR: Adjusted odds ratio
CBHI: Community-based health insurance
CI: Confidence interval
ETB: Ethiopian birr
HI: Health insurance
OOP: Out of pocket
SHI: Social health insurance
UHC: Universal health coverage
WTP: Willingness to pay.
